# Automatic Lumen Detection on Longitudinal Ultrasound B-Mode Images of the Carotid Using Phase Symmetry

**DOI:** 10.3390/s16030350

**Published:** 2016-03-09

**Authors:** José Rouco, Elsa Azevedo, Aurélio Campilho

**Affiliations:** 1 INESC TEC – INESC Technology and Science, FEUP, Rua Dr. Roberto Frias s/n, Porto 4200-465, Portugal; campilho@fe.up.pt; 2 FMUP – Faculty of Medicine, University of Porto, Porto 4200-319, Portugal; eazevedo@med.up.pt; 3 Department of Neurology, São João Hospital Center, Porto 4200-319, Portugal; 4 FEUP – Faculty of Engineering, University of Porto, Porto 4200-465, Portugal

**Keywords:** carotid ultrasound, lumen detection, intima-media thickness

## Abstract

This article describes a method that improves the performance of previous approaches for the automatic detection of the common carotid artery (CCA) lumen centerline on longitudinal B-mode ultrasound images. We propose to detect several lumen centerline candidates using local symmetry analysis based on local phase information of dark structures at an appropriate scale. These candidates are analyzed with selection mechanisms that use symmetry, contrast or intensity features in combination with position-based heuristics. Several experimental results are provided to evaluate the robustness and performance of the proposed method in comparison with previous approaches. These results lead to the conclusion that our proposal is robust to noise, lumen artifacts, contrast variations and that is able to deal with the presence of CCA-like structures, significantly improving the performance of our previous approach, from 87.5%±0.7% of correct detections to 98.3%±0.3% in a set of 200 images.

## 1. Introduction

Atherosclerosis is a disease of the vessel walls that can lead to serious cardiovascular accidents, like myocardial infarction and stroke. B-mode ultrasonography of the arteries, such as the extra-cranial portion of the carotid, is widely used in clinical practice, as it is a non-invasive, low cost and readily available medical imaging technology. It is a preferable modality for the visualization of the anatomical layers of vessel walls, allowing the measurement of the intima-media thickness (IMT), the lumen diameter or the identification of atherosclerotic plaques in the common carotid artery (CCA) [1,2]. The IMT measurement is being increasingly used as a surrogate marker of atherosclerosis, showing the capability to predict the risk of clinical vascular events [3,4,5,6].

Most of the existing IMT measurement methods require user interaction to locate a region of interest (ROI), containing the targeted structures for analysis [7]. Other algorithms, usually referred to as fully-automatic, incorporate a CCA detection algorithm as a previous step for ROI location [8,9,10,11,12,13,14,15,16]. The objective of this CCA detection step is to locate the vessel structure within the image, so that the posterior IMT segmentation and measurement does not get confused with other similar structures. This work is focused on this stage with the objective of deriving a robust method for CCA location. Concretely, the CCA will be located through the detection of a path going through the lumen region, as required by the IMT segmentation methods presented in [9,17]. Additionally, the proposed detector is designed to locate paths that are near the lumen centerline.

In the ultrasound images, the CCA lumen appears as an oriented, nearly horizontal low echogenicity region surrounded by two layered bright bands corresponding to the artery walls [10]. The shape of the CCA regions varies with the patient anatomy and with the imaging plane. Furthermore, it can be affected by the presence of atherosclerotic plaques. Other anatomical structures similar to the CCA shape, such as the jugular vein or the sternocleidomastoid muscle, may mislead the detection strategies [9,13,14,15,18].

In addition, ultrasound images are characterized by the presence of speckle noise and local changes of intensity and contrast. Some common consensus on carotid IMT measurement includes guidelines for B-mode image acquisition [1,2]. These recommendation guidelines include references to the control of the overall gain to reduce lumen noise and to adapt the time gain compensation to achieve similar brightness on both artery walls. This is one of the reasons why some intensity-based methods have been successfully used for CCA detection [9,11], regardless of not being well suited for ultrasound image analysis. Nevertheless, these ultrasound settings are ultimately established according to the subjective judgment of the operator. Thus, robust methods for CCA detection should be able to deal with varying CCA shape, the presence of varying anatomical structures, the presence of lumen artifacts and local intensity and contrast changes.

### 1.1. Related Work

For automatic IMT measurement, CCA detection and ROI selection are often approached by heuristic methods without analysis or discussion of the method limitations and the parameter settings. Only a few papers are focused on the topic of CCA detection. Rossi *et al.* [10] propose a method for lumen centerline detection on ultrasound video sequences, which is robust to noise and gain setting variations, but is sensible to a low signal-to-noise ratio (SNR) and to the presence of long segments of jugular vein. Molinari *et al.* [11] proposed a method to detect both CCA lumen and walls, obtaining good detection performance on images acquired with different ultrasonographers and only reporting a few misdetections for high levels of lumen noise and artifacts. In [18], we presented an approach for lumen centerline detection that achieved good performance under varying levels of Gaussian and speckle noise. However, the method might result in false CCA detections due to confusion with other structures of similar shape. In a recent paper, Rocha *et al.* [19] presented an independently-developed approach to CCA detection that proposes a solution for the detection of multiple candidates.

The existent CCA detection algorithms follows two main strategies. The first is a column-wise analysis to locate and connect candidate points to create line segments corresponding to the CCA lumen or walls [10,11,12,13,14,15], where the longest candidate segment is usually selected as corresponding to the CCA. The second strategy uses a dynamic programming (DP) algorithm to find a path, from one image border to the other, optimizing a global cost or gain function that weights relevant features of the CCA structures [8,9,18,19]. The research herein described falls into this second DP-based strategy.

Rocha *et al.* [9] proposed to detect the CCA lumen centerline using DP to connect lumen symmetry axis points derived from the thresholding segmentation of the lumen region. The lumen symmetry information was also optimized with DP for lumen centerline location in our previous approach [18]. Using a contrast-invariant symmetry measure that does not depend on previous segmentation improved the robustness to contrast changes and high levels of lumen noise. In [19], Rocha *et al.* use DP to locate the far wall contours and the lumen centerline candidates through the connection of local maxima of Gaussian derivatives and local minima of Gaussian smoothed images, respectively. The misdetection of other symmetric structures in [18] motivated additional research, described in this paper. Rocha *et al.* [19] solved this issue in their recent work by adding a classification stage to the DP-based candidate detection stage.

The most important differences among the previous CCA detection methods are on the criteria and features used. Some use criteria based on vessel wall features, such as the local shape, the contrast or the width of the vessel wall structures [10,11,12,13,19]. Other approaches use lumen region features, such as the average echogenicity [8,9,11,15,19], gray level variability [10,11] or lumen diameter [9,10,18].

However, under imaging conditions where similar structures appear, misdetections can occur regardless of the criterion. Some of the previous approaches proposed heuristic strategies [9,14,15,19], assuming that the CCA appears below, which are most likely to correspond to the sternocleidomastoid muscle or the jugular vein. In these references, the common approach consists of selecting the bottom-most among a list of good enough candidates according to a certain criterion, instead of simply selecting the best fitting one. In this paper, a procedure inspired by this idea is described.

### 1.2. Proposed Work

The goal of the work herein described is to improve the detection results of the symmetry-based method in [18], by introducing advanced selection mechanisms that deal with the lumen misdetection among similar image structures. This advanced selection mechanism is the major contribution of this approach. Instead of selecting the DP path with absolute maximum accumulated symmetry, all of the local maxima paths in the DP profile are detected as potential lumen centerline candidates. Then, these candidates are evaluated using different symmetry-, contrast- and intensity-based classification, so that a refined set of candidates is identified. Finally, the CCA lumen centerline is selected among these candidates using a position-based heuristic, by selecting the bottom-most candidate.

The proposed method is different from [9], as it avoids the segmentation step by a robust symmetry-based detector similar to that in [18]. It is also different from [18], as it includes an advanced selection procedure that faces the presence of multiple candidates. The differences with the recent paper [19] are the parameter adaptability and a better generalization capability, supported by in-depth experimental methodology. In fact, the proposed method uses the simpler robust approach in [18] for candidate detection, with parameters that can be directly estimated from the domain knowledge of the CCA radii. Second, the classification features are different from those of the detection stage, generalizing the approach and allowing one to incorporate additional information through any set of generic features. Finally, the experimental methodology of the present study is more complete, allowing one to evaluate which features are responsible for the correct detection of the CCA.

Several experimental results are provided to evaluate the performance and robustness of the method using symmetry, intensity or contrast features. Additionally, unlike many of the previous works, the parameter settings, the method limitations and the possible future improvements are discussed in detail.

## 2. Automatic Lumen Detection Method

**Figure 1:**
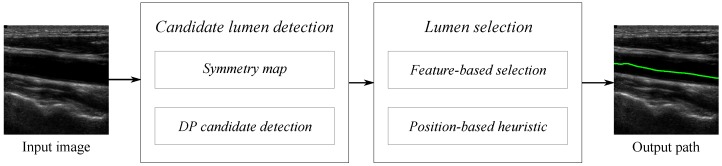
Diagram of the different stages in the proposed automatic lumen detection method.

This section describes a method for the automatic detection of the CCA lumen centerline from longitudinal B-mode ultrasound images. Our proposal is organized in the different stages summarized in the scheme of Figure 1 and detailed below.

First, the input B-mode image is processed to compute a symmetry map using local phase symmetry analysis, as described in Section 2.1 and in the Appendix. In the next stage, a set of potential lumen centerline candidates is detected using the symmetry map with a modification of the lumen detection in [18], as presented in Section 2.2. Intensity, symmetry and two lumen-to-wall contrast maps are the features extracted from the input image on each one of the detected lumen candidates. These features are defined in Section 2.3. At the last stage, the lumen is selected among the candidates as described in detail in Section 2.4. The candidates are first classified using the CCA features, and the final CCA lumen centerline is selected among a set of candidates using a position-based heuristic.

### 2.1. Symmetry Map

Our previous method for CCA detection [18] improved the detection results of [9] by substituting the segmentation step by the computation of a symmetry map using local phase analysis of patterns at an appropriate scale. This symmetry map, denoted as $Sym(\vec{x})$, is also used as one of the cues for CCA detection in this work. The local phase information is computed using monogenic signal analysis [20] applied to a band-pass-filtered version of the input B-mode image. The parameters controlling the band-pass filter allow one to limit the phase analysis to a range of scales of interest. As in [18], it is assumed that the possible CCA diameters are in the interval $\left[D_{min},D_{max}\right]$, and the filter parameters are adjusted accordingly. The Appendix provides detailed information about the computation of the symmetry map from the input B-mode image. The estimation of the diameter parameters is discussed in detail in Section 3.2.

### 2.2. Candidate Lumen Detection

The CCA detection method in [18] used a DP process to select the path that optimizes the symmetry map described in Section 2.1. The DP algorithm was used to find the path P, connecting the left and right image borders, that maximizes a gain function *G* that integrates the local symmetry at the path positions:

(1) $$\begin{array}{cc} & G\left(P\right)=\sum_{t=1}^{N}Sym(p_{t})\\ \text{subject}\;\text{to}:\; & p_{t}⋈p_{t+1}\;;\;t=1,\cdots ,N-1 \end{array}$$

where $P=(p_{1},p_{2},\cdots ,p_{N})$ denotes a path passing through image positions $p_{t}={\left(t,y_{t}\right)}^{⊤}$ for each of the *N* image columns and $p_{t}⋈p_{t+1}$ is a relation meaning that the two points are connected, which in this case corresponds to $|y_{t}-y_{t+1}|\leq 1$ in order to ensure eight-connectivity.

**Figure 2:**
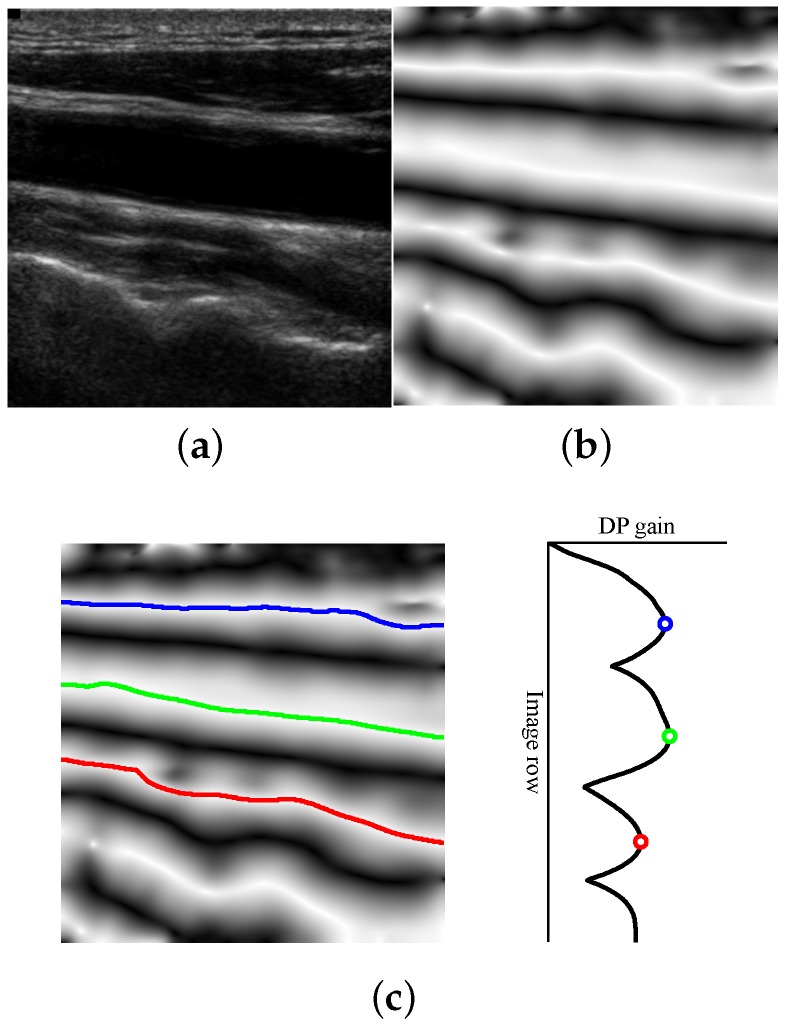
Illustration of the candidate lumen detection algorithm. (**a**) Input image; (**b**) symmetry map; (**c**) lumen candidates in the image and in the dynamic programming (DP) gain profile.

The DP algorithm propagates the gain function of Equation (1) from the left to the right image borders using the following recursive form:

(2) $$\left\{\begin{array}{ccc} G^{*}\left(p_{n}\right) & = & \max_{p_{n-1}⋈p_{n}}\left\{G^{*}\left(p_{n-1}\right)\right\}+Sym\left(p_{n}\right)\\ G^{*}\left(p_{1}\right) & = & Sym(p_{1}) \end{array}\right.$$

where $G^{*}\left(p_{n}\right)$ is the gain of the optimal path ending at each $p_{n}$, corresponding to each row at the *n*-th column of the image. In addition to the optimal gain, the best connection is saved for each $p_{n}$. Thus, from each position, the optimal path can be found by tracking back these connections. At the last column, $G^{*}\left(p_{N}\right)$ is the DP gain profile that qualifies the different paths that are available for selection, for each image row. An example of this profile and its corresponding candidates is depicted in Figure 2c.

In [18], the path with the best gain in $G^{*}\left(p_{N}\right)$ was selected as the lumen centerline. This optimization of the symmetry map is robust against high contrast variations and lumen artifacts, as it detects the overall symmetric shape of the dark structures. However, there may be other significant structures with a similar shape to the carotid artery (e.g., the sternocleidomastoid muscle or the jugular vein) that also correspond to higher values on the symmetry map, leading to misdetections, when using the best DP gain selection strategy.

A more advanced candidate selection strategy is proposed here in order to avoid these misdetections. The idea is to select a few candidates from the DP optimization process and analyze them in detail to perform the final lumen selection as described in Section 2.4.

The candidate lumen detection strategy relies on the detection of significant local maxima in the DP gain profile $G^{*}\left(p_{N}\right)$. These local maxima correspond to the main symmetric dark structures, as illustrated in the example of Figure 2. In order to compute these local maxima, a first order Gaussian derivative filter is applied to the DP gain profile. The standard deviation parameter of the Gaussian filter, denoted here as $\sigma_{peak}$, allows the control of the scale of analysis. The positive slope zero crossings are detected in the resulting profile by evaluating positions $\pm \sigma_{peak}/2$ apart from each point. This may result in the duplicate local maxima detections in adjacent positions, which can be further removed by selecting the point with maximum gain for each set of adjacent peaks.

### 2.3. Features

The detected lumen centerline candidates are evaluated using different feature-based criteria. The overall image appearance of the CCA is characterized by a tubular shape, a low lumen echogenicity and a high echogenicity of the adventitia layers at the artery walls. In order to select appropriate lumen centerline positions characterized by these properties, we measure the symmetry, lumen intensity and lumen-to-wall contrast.

- Symmetry: The CCA shape is evaluated using the symmetry measure Sym(x) of Equation (A1), which is appropriate for the detection of tubular dark structures surrounded by brighter regions, giving rise to lumen centerline candidates that go through positions of high symmetry. As this measure only depends on the local phase, it is independent of the contrast and intensity.
- Intensity: The low echogenicity of the lumen region is evaluated directly from the input image I(x). Ultrasound imaging parameters are usually adjusted for the lumen region to appear as a homogeneous dark area. Thus, an appropriate lumen centerline candidate should go through positions of low image intensity.
- Contrast: The adventitia regions are lighter than the lumen region, and the lumen-adventitia contrast is usually the highest in the overall image. According to this, appropriate lumen centerline candidates should go through positions where the intensity difference with nearby adventitia positions is high. These intensity differences are estimated by the contrast measures $C^{+}\left(x\right)$ and $C^{-}\left(x\right)$ defined below, in Equation (3).

The CCA structure is characterized by a high lumen-to-wall contrast. In order to evaluate the lumen centerline positions, we propose to roughly estimate the contrast using two difference of offset Gaussian (DooG) contrast maps that provide an estimation of the lumen-to-wall intensity differences at each lumen centerline point. The relative location of the adventitia regions with respect to each lumen centerline point is not known. Instead, it is assumed that the carotid arteries have about the same width for all of the images, so that the distance from a lumen centerline position to the nearest artery wall position is approximately located at a fixed vertical distance.

The lumen-to-wall contrast $C^{\pm }$ maps are computed using DooG filters as follows:

(3) $$C^{\pm }\left(x\right)\;=\;G_{off}\left(x\pm \delta \Delta_{y}\right)-G_{on}\left(x\right)$$

where x denote the image position, $G_{on}$ and $G_{off}$ are the two different Gaussian smoothings of the input image with standard deviations of $\sigma_{on}$ and $\sigma_{off}$. $\Delta_{y}={\left(0,1\right)}^{⊤}$ is used to vertically displace the $G_{off}$ Gaussian by a given offset *δ*, which depends on the average CCA radius. The smoothing scales $\sigma_{on}$ and $\sigma_{off}$ depend on the width of the lumen and adventitia regions, respectively. The estimation of these parameters is discussed in detail in Section 3.2.

Two different contrast maps are considered: one measures the contrast on the near wall of the artery and the other measures the contrast on the far wall, denoted by $C^{-}\left(x\right)$ and $C^{+}\left(x\right)$, corresponding to the negative and positive offsets, respectively.

**Figure 3:**
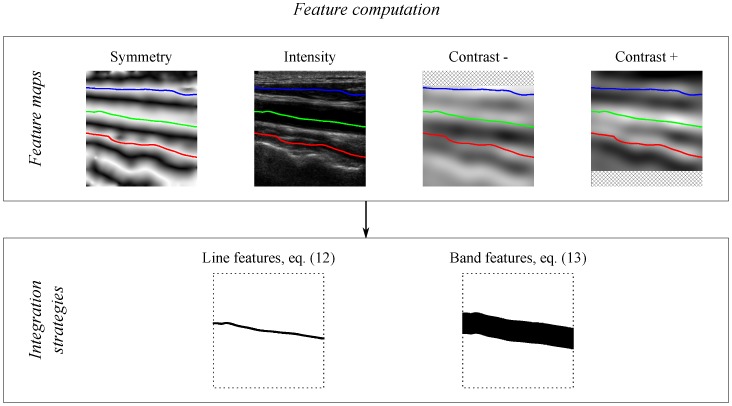
Feature computation. The symmetry Sym(x), intensity I(x) and contrast $C^{\pm }\left(x\right)$ feature maps are integrated along the candidate lumen paths using line and band strategies.

The symmetry Sym(x), the intensity I(x) and the contrast $C^{\pm }\left(x\right)$ feature maps give information on each image position x. In order to obtain features associated with each candidate lumen centerline path, these map positions are combined using two alternative integration strategies, on the path line and on a band region around the path, as illustrated in Figure 3.

The line features are computed from the candidate path positions as:

(4) $$F_{M}^{C}\left(P\right)=\frac{1}{N}\sum_{t=1}^{N}M(p_{t})$$

where $p_{t}$ are the positions of the path P and M is one of the previously-described feature maps.

The band features are computed from a band region within a vertical radius $R_{f}$ from the candidate path positions as:

(5) $$F_{M}^{R}\left(P\right)=\frac{1}{2R_{f}+1}\sum_{r=-Rf}^{R_{f}}F_{M}^{C}\left(P+r\Delta_{y}\right)$$

where $\Delta_{y}={\left(0,1\right)}^{⊤}$ is used to vertically displace the positions of the path P by *r* and M is a feature map.

It should be noted that in the case of the contrast maps $C^{-}$ and $C^{+}$, there is a band region, above and below, respectively, where this computation is not possible due to the offset displacement of the $G_{off}$ falling outside of the image. In order to avoid the contrast features being indeterminate for some candidates, the candidates that completely fall at a lower distance than *δ* from the upper and bottom borders of the image are discarded.

### 2.4. Lumen Selection

The features in Section 2.3 should be good indicators for the CCA lumen centerline candidates. However, under the specific imaging conditions, some other candidates corresponding to other anatomical structures result in similar features to those of the carotid artery. Instead of solely basing the lumen selection on the feature values, we add some domain information, corresponding to the relative anatomical location of CCA in relation to other similar anatomical structures. These two sources of information are combined in sequence by performing a feature-based selection stage followed by a position-based selection stage.

At the feature-based selection stage, the candidate paths are evaluated on the basis of the features described in Section 2.3. The feature-based selection is performed using a one-dimensional logistic classifier [21]. The goal of this classifier is to detect the candidates that resemble the CCA. Each one of the individual features is used in a different classifier with the aim of comparing their performance on lumen candidate evaluation. The combination of several features in a single classifier is not considered as the individual features achieved high performance, as is reported in Section 4.2. The contrast and symmetry features are normalized by subtracting the maximum value of all of the candidates in an image. The intensity feature is normalized subtracting the minimum. This way, for each image, the features are relative to the best candidate.

In the position-based selection stage, the basic assumption is that the CCA appears below any other of the similar structures, often corresponding to the sternocleidomastoid muscle or the jugular vein. Thus, the bottom-most of these candidates is selected as the lumen.

The logistic classifier is trained in the following way. From a set of training images, the lumen candidates are detected as described in Section 2.2. For each one of these candidates, the features are computed according to Section 2.3 and normalized. The true CCAs are labeled as positive class; the candidates below the true CCAs are labeled as negative. Note that we are not including the candidates above the CCA as negative samples, because some of them may be similar to the CCA and would introduce noise in the training phase. Thus, the feature-based classification stage is only focused on differentiating the CCA from any possible candidate structures appearing below it.

## 3. Materials and Experimental Methods

This section describes the experimental methodology used to test the CCA lumen centerline detection method. The experiments described here include the evaluation of the candidate detection method and the performance comparison of the candidate selection with the different features. These experiments use a dataset of 200 images (Section 3.1). The parameter settings are discussed in Section 3.2. In addition, the experimentation includes an analysis of robustness under several levels of Gaussian and speckle noise (Section 3.3) and the comparison of the proposed method against other methods (Section 3.4).

### 3.1. Image Database

An image dataset of a total of 200 longitudinal B-mode CCA images was used. Fifty of these images correspond to the dataset used in [9,17,18], which was acquired at Hospital São João in Porto (Portugal). This dataset was taken from 25 different symptomatic patients. The remaining 150 images were provided by courtesy of Dr. Filipo Molinari, from University of Torino (Italy). It is a subset of the 200 image dataset used in [12,14], originally acquired from 150 patients. Both sets of images were acquired with ATL-HDI5000 ultrasound systems. The ultrasound parameters were set by the ultrasound technicians, which followed the acquisition protocols used in their hospital departments. The more noticeable differences in the parameter settings of the two datasets is on the choices of the time gain compensation (TGC), as the images on the former dataset are quite attenuated below the CCA, while the same areas on the latter dataset are clearly gain-compensated. In addition, 28 images of the first dataset and 11 of the second contain plaques. The images were resampled using linear interpolation, so that the pixel size was normalized to 0.09 mm as in [9,17]. However, the proposed algorithms are not sensible to such normalization if the scale parameters are varied according to the pixel size, as the main processing steps rely on near-CCA scale information by filtering the image details at lower scales than the minimum lumen diameter. To that end, the parameter settings derived in Section 3.2 are expressed in mm.

The CCA intima-media regions were manually segmented by medical experts for the 200 images. They delineated the boundaries between the lumen and the intima (LI) and between the media and the adventitia (MA), for both the near and far CCA walls. These contours are used to evaluate the lumen centerline detection method by taking into account that the candidate path points fall between the near and far wall LI boundaries. This guarantees that the resulting paths go through the lumen, avoiding the plaques.

The images were divided into training and test sets with randomized *K*-fold cross-validation. The images in the training sets were used to train the feature-based selection, as described in Section 2.4 The test set images, with added noise as described in Section 3.3, are used to estimate the method performance. The number of folds was set to K=5 to obtain diverse enough test sets, as there are several artifacts and exceptional issues involved in automatic CCA detection. The experiments were repeated 50 times to deal with the randomizing effects of the *K*-fold dataset division and the noise simulation.

### 3.2. Parameter Settings

**Figure 4:**
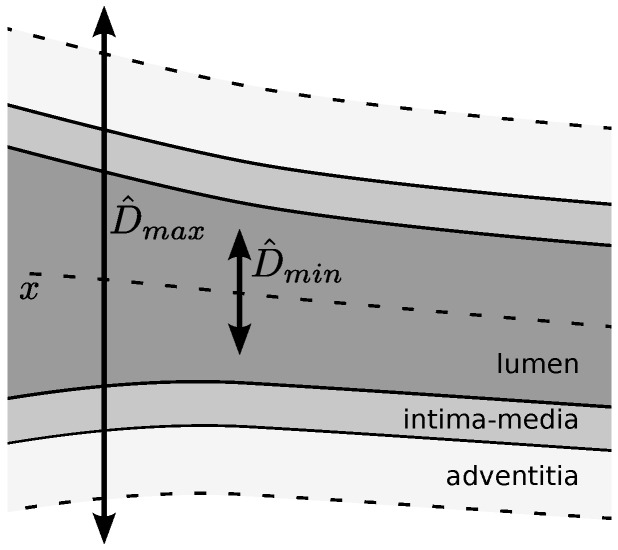
Scheme depicting the estimated diameter measures.

All of the parameters are related to the scale of the structures of interest and are suitable for domain knowledge heuristic settings. For this purpose, we use a rough estimate of the minimum CCA internal diameter ${\hat{D}}_{min}$, which excludes the artery wall, and a rough estimate of the maximum CCA outer diameter ${\hat{D}}_{max}$, which includes the brightest regions of the adventitia, as depicted in Figure 4. The used values for these estimates are derived from population studies and consensus statements [1,2,22].

Krejza *et al*. [22] reported the mean CCA internal diameters for 500 patients in a plaque-free zone. The CCA internal diameters in women (6.10±0.80 mm) are significantly smaller than in men (6.52±0.98 mm); however, the difference is much smaller than the diameter variation within the same gender, as influenced by other factors, like body and neck size, age and blood pressure. Furthermore, in women, the left CCA diameters (6.05±0.87 mm) are slightly smaller than the right CCA diameters (6.15±0.91 mm). This was not the case in men. Using these data, we estimate ${\hat{D}}_{min}$ as the lower bound of the 99% confidence interval for the left CCA diameters in women, resulting in ∼4.03 mm.

On the other hand, the upper bound of the 99% confidence interval for the CCA diameters in men is used to estimate the maximum CCA internal diameter, resulting in ∼8.80 mm. This measure is increased to estimate ${\hat{D}}_{max}$ by adding the width of both artery walls, which includes the intima-media thickness (IMT) and the width of the adventitia regions. The average IMT in plaque-free regions varies with age and gender, among other factors [2]. Current consensus established that an IMT larger than 1.5 mm can be considered as a plaque [1]; thus, we use this value as an upper bound for the intima-media complex in plaque-free regions. Finally, in order to include the echogenic regions of the adventitia, for which population studies are not available, we consider an extra wall width of 2 mm, resulting in a total ${\hat{D}}_{max}$ of 15.80 mm.

From the CCA diameter range $\left[{\hat{D}}_{min},{\hat{D}}_{max}\right]$, the other parameters are set as described below. Table 1 shows a summary of each of the parameter estimation expressions and the resulting values.

#### 3.2.1. Symmetry Map

The computation of the symmetry measure uses two parameters, $D_{min}$ and $D_{max}$, roughly corresponding to the minimum and maximum CCA diameters. These parameters control the soft cut-off frequencies of the band-pass filters, for selecting the scales of analysis; thus, we set their values to the estimated ${\hat{D}}_{min}$ and ${\hat{D}}_{max}$. In [18], these parameters were roughly estimated as the absolute minimum intima-intima and maximum of adventitia-adventitia diameters for the used dataset, which correspond to 4.12 mm and 13.65 mm, respectively. However, it was experimentally concluded that increasing the band-width of the band-pass filter benefits the lumen centerline location in some cases, and varying the diameter range by several millimeters does not affect the detection results.

#### 3.2.2. Peak Detection

$\sigma_{peak}$ was defined as the scale of the Gaussian derivative for peak detection in the DP profile. This scale of analysis is fixed to $\sigma_{peak}={\hat{D}}_{min}/4$, which is a high enough value to avoid the selection of noisy peaks that may occur due to lumen artifacts. It should be noted, however, that a much higher value could be used without preventing the algorithm from detecting the relevant peaks, which, in the worst case, should at least be spaced by a much larger distance than ${\hat{D}}_{min}$.

#### 3.2.3. Contrast Maps

**Figure 5:**
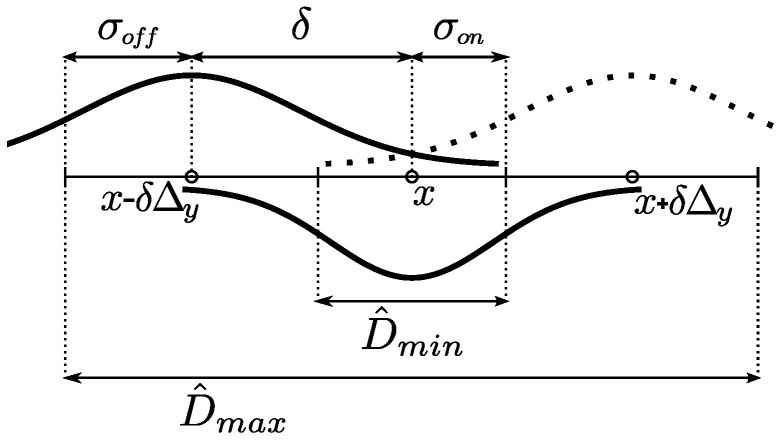
Estimation of the difference of offset Gaussian (DooG) parameters values for the measurement of lumen-to-wall contrast.

The parameters $\sigma_{on}$, $\sigma_{off}$ and the offset *δ* were defined to adjust the DooG filter parameters, used to measure lumen-to-wall contrast. These parameters correspond to the lumen region radius, the wall region radius and the expected distance between the centerlines of the two regions. Figure 5 depicts a scheme relating these parameters with the maximum and minimum CCA diameters. For the lumen region radius $\sigma_{on}$, half of the minimum CCA diameter ${\hat{D}}_{min}$ is used, so that it can be expected that only low echogenicity values of the lumen region are averaged. The artery walls should be located in the remaining regions between the minimum and maximum diameters on both sides of the lumen centerline. Thus, the $\sigma_{off}$ is adjusted to half the width of these wall regions and the *δ* offset to the distance from the lumen centerline to the center of these regions, following the expressions in Table 1.

#### 3.2.4. Band Features

Finally, the radius $R_{f}$ around the candidate paths is set equivalent to the $\sigma_{on}$ radius, so that the features are computed for the positions corresponding to the lumen region.

### 3.3. Noise Models

In order to test the robustness of the method, artificial noise is added to the input test set images *I*. The two models proposed in the experimentation of [18] are used. The first model corresponds to additive speckle noise following the equation:

(6) $$\begin{array}{ccc} I_{S} & = & I+I^{\gamma }n \end{array}$$

where $n∼N(0,\sigma^{2})$ is the normally-distributed noise with zero average and standard deviation *σ* and *γ* is a parameter that depends on the post-processing of the ultrasound signal for the image formation [23]. It has been shown that this model adjusts to the speckle noise in log-compressed ultrasound images with γ=1/2 [24,25].

The artificially-generated multiplicative speckle noise is weak in dark image areas, such as the lumen region, indicating that speckle is not the main source of lumen noise, which could affect the CCA detection methods. Most of the previous approaches report the influence of non-uniform lumen backgrounds, lumen artifacts and poor contrast regions. However, as the ultrasound operator usually adjusts the dynamic range and gain parameters to make the lumen region appear as dark and uniform as possible [1,2], the image dataset does not contain many examples of these situations. In order to simulate and evaluate the effect of these artifacts, we use additive white Gaussian noise as a second model, following the equation:

(7) $$\begin{array}{ccc} I_{G} & = & I+n \end{array}$$

where $n∼N(0,\sigma^{2})$ is the normally-distributed noise with zero average and standard deviation *σ*.

**Figure 6:**
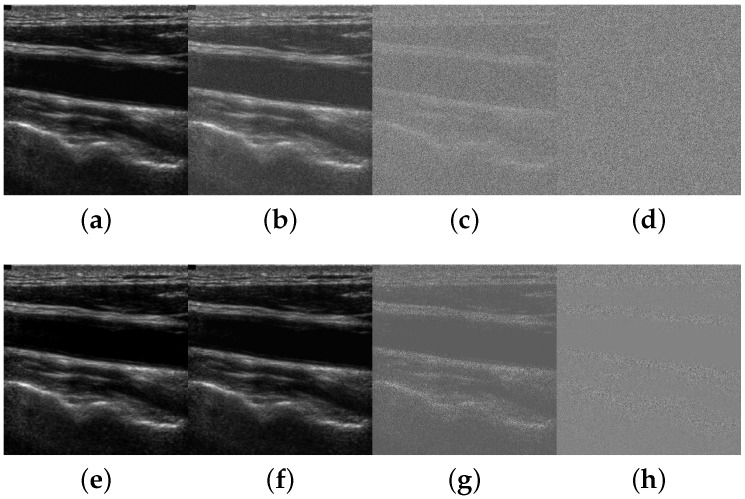
Examples of carotid ultrasound images with different levels of Gaussian (first row) and speckle (second row) noise. (**a**) 30 dB; (**b**) 10 dB; (**c**) -10 dB; (**d**) -30 dB; (**e**) 30 dB; (**f**) 10 dB; (**g**) -10 dB; (**h**) -30 dB.

Figure 6 depicts a carotid ultrasound image example that has been subject to different noise levels following the two models.

The signal-to-noise ratio (SNR) varies from 30 dB to -30 dB. Although noise levels from 0 dB to -30 dB are not common, they were included in the experiments to assess the noise robustness limits in challenging situations.

### 3.4. Methods Used for Comparison

The lack of appropriate experimental evaluation, the lack of adequate algorithm details and the lack of standardized databases do not ease the comparison of our method with others. In this work, however, two alternative lumen centerline detection methods, denoted as Best-*Sym* and Bottom-most, were used for comparison purposes.

The Best-*Sym* method uses the symmetry map described in Section 2.1 and a DP optimization as described in Section 2.2 to select the lumen centerline that achieves the maximum aggregated symmetry value in the DP gain profile. It corresponds to the method in [18], which was compared to the CCA detection algorithm in [9] using the 50 image dataset from Hospital São João. The reported results have shown that the work in [18] outperformed [9] in contrast variation and noise robustness. Best-*Sym* is now compared to the method herein proposed using a larger and more complex image dataset.

The method proposed in this paper is different from Best-*Sym* in three main aspects: (1) it allows one to complement the symmetry-based criterion with intensity- or contrast-based criteria; (2) it adds a feature-based candidate selection approach for classification; and (3) it incorporates a position-based heuristic. The performance of this method depends on the combined performance of the candidate detector, the classifier and the position-based heuristic, while the performance of the Best-*Sym* method depends on a symmetry-based greedy detection.

The Bottom-most method consists of applying the position-based heuristic to the results of the candidate lumen detector described in Section 2.2, without performing any feature-based classification. It may have happened that the candidate detector was good enough to not need the feature-based classification step, so that the selection of the bottom-most candidate was enough for CCA detection. This method is included for comparison purposes in order to quantify the improvement obtained by adding the feature-based classification step to the position-based heuristic.

## 4. Results

### 4.1. Candidate Lumen Detection

**Figure 7:**
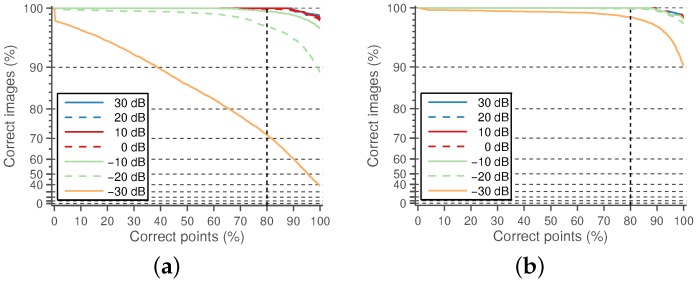
Performance of the candidate lumen detection algorithm as a percentage of images with the correct candidate depending on the percentage of correct points of the candidate. (**a**) With added Gaussian noise; (**b**) with added speckle noise. The curves corresponding to SNRs under 0 dB are mostly overlapped.

A detected CCA lumen centerline is considered a correct detection if the path points fall between the near and far wall LI boundaries defined by the experts. It is considered wrong otherwise. The performance of the candidate lumen detection algorithm is measured as the percentage of images in which at least one of the detected candidates is correct. These results, under varying levels of Gaussian and speckle noise, are shown in Figure 7. The experiments are repeated 50 times, and the mean performance is depicted.

**Figure 8:**
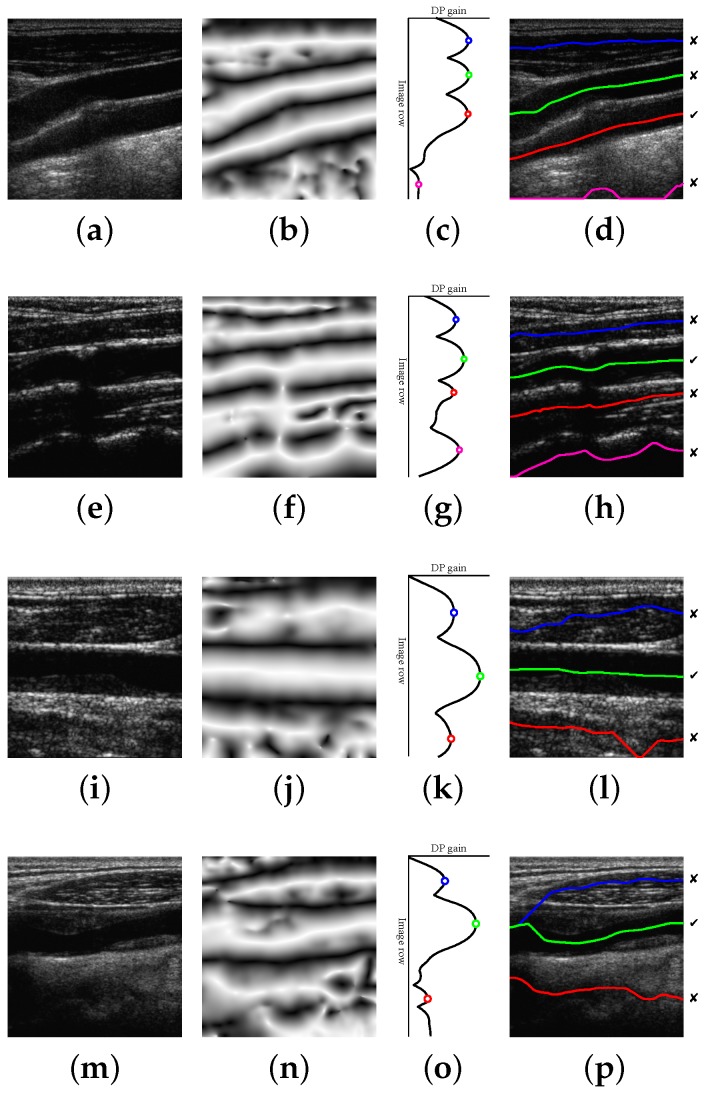
Candidate lumen detection examples. From left to right: (**a**,**e**,**i**,**m**) input image; (**b**,**f**,**j**,**n**) symmetry map; (**c**,**g**,**k**,**o**) DP gain profile; and (**d**,**h**,**l**,**p**) resulting candidates, indicating which one is closer to the true lumen (the one marked with ✓). Images (**a**) and (**e**) result in candidate detections with 100% of correct points. Images (**i**) and (**m**) result in candidate detections slightly touching the plaque boundaries: (**l**) at the image center and (**p**) at the left of the image.

The proposed method is able to detect a candidate with 100% of the correct points in 196 images out of 200. This high performance is maintained for a reasonably high level of Gaussian and speckle added noise. In the remaining four images, the misdetection is due to approximately 10% of path points, on average, lying outside the lumen region. Some candidate detection examples are shown in Figure 8. The examples of Figure 8a,e correspond to 100% correct lumen centerline candidate detection. The examples of Figure 8i,m are typical cases of candidate lumen centerline detections with few misdetected points. In Figure 8i, the best candidate touches a low contrast plaque at the image center. In Figure 8m, the best candidate touches the LI boundary at the left part of the image due to the bad quality imaging of the nearby left area.

**Figure 9:**
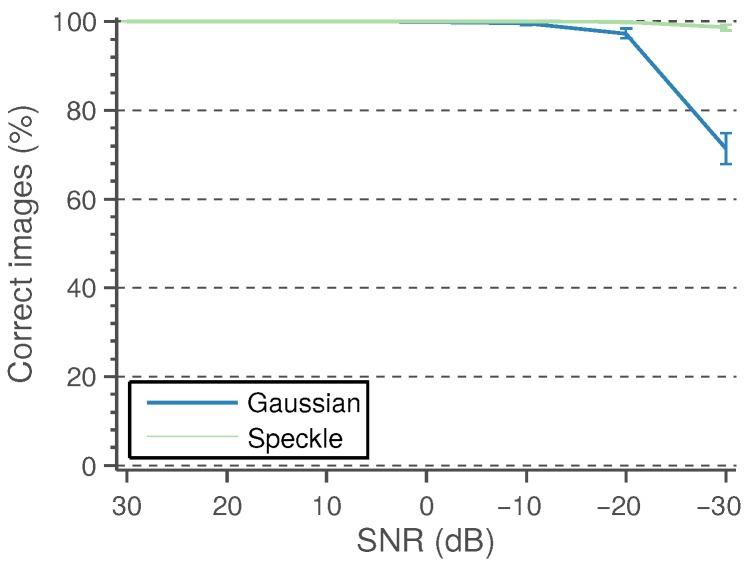
Performance of the candidate lumen detection algorithm as a percentage of images with 80% correct candidates depending on the noise levels.

A small number of misdetected points should not be important, as the main objective of this work is to approximately locate the CCA in the image for further accurate processing. Even more, it was manually verified that the incorrectly-detected points are not severely misplaced with respect to the lumen region. For this reason, it was considered that a candidate path was correctly detected if at least 80% of its points fell within the near and far wall expert LI boundaries. The detection results with this criterion are shown in Figure 9. It can be observed that the detection method is only unable to detect an appropriate lumen candidate for a few cases, mainly for cases with high noise levels.

Each test case where the candidate lumen detection algorithm was not able to correctly detect the true lumen within their candidates was removed from the following processing steps. Thus, the reported performances of the subsequent stages are independent of the performance of the candidate detection algorithm, which should be taken into account to assess the overall method performance. In fact, only a few cases of high noise levels were removed from the experiments using the “80% correct” criterion.

### 4.2. Feature-Based Selection

The values of the eight features in Section 2.3 are computed for the true and false lumen candidates considered for the classification stage. The feature values are summarized in Table 2.

**Figure 10:**
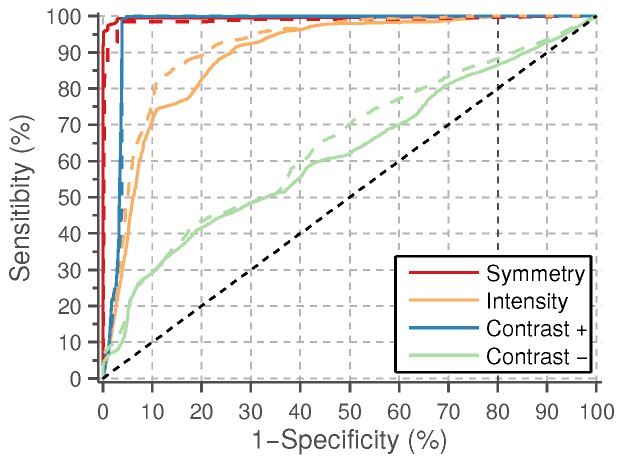
Receiver operating characteristic (ROC) curves for the eight one-dimensional classifiers used for feature-based lumen candidate selection. Solid lines represent line features. Dashed lines represent band features.

**Figure 11:**
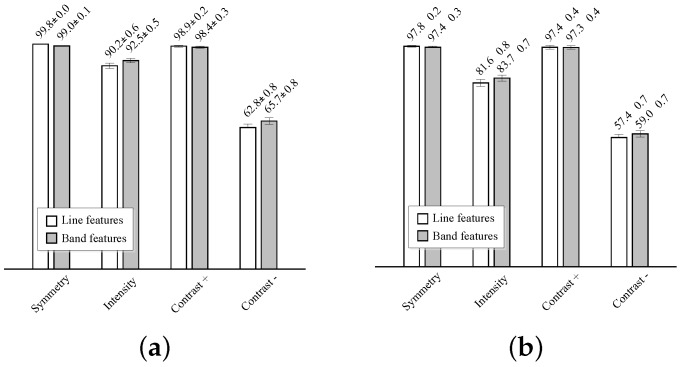
Performance measures for the eight one-dimensional classifiers used for feature-based candidate lumen selection. (**a**) Average area under the curve (AUC); (**b**) average best accuracy.

The one-dimensional logistic classifiers, described in Section 2.4, are trained using each one of the eight features, with equal *a priori* probabilities for the two classes. The average classifier performances on the test sets are reported using the receiver operating characteristic (ROC) curves depicted in Figure 10. The average area under the curve (AUC) and average best accuracy are compared in Figure 11.

The best results are obtained by the symmetry and positive contrast (differences with the far wall regions), either using line or band features. A slightly higher classification performance is obtained with the line features. The performance of these individual features is close to 100%, thus more complex models for classification, like, e.g., those combining several of these features, are not needed. The intensity features have a significantly lower performance than the symmetry and contrast, and a very poor accuracy is obtained with the negative contrast features, corresponding to the lumen intensity differences on the near wall regions.

### 4.3. Automatic Lumen Detection

**Figure 12:**
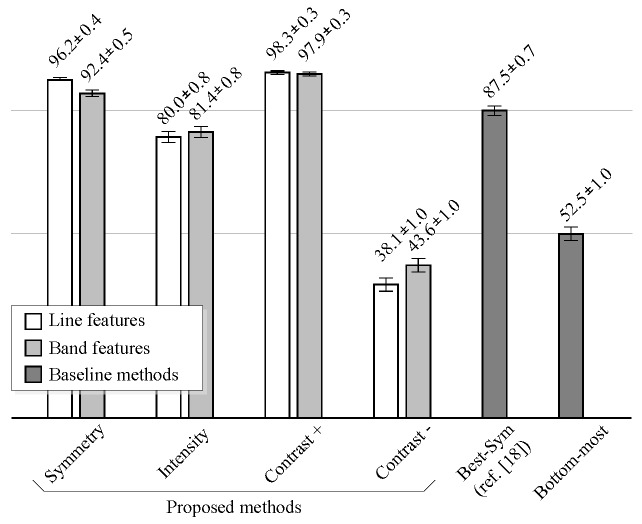
Comparison of lumen detection results in % of images. The proposed methods are compared to the baseline methods in noiseless images.

The performance of the method depends on the individual performances of the candidate detector, of the feature-based candidate selection and on the number of conflicting candidates per image and their relative position. Figure 12 shows the performance comparison of the proposed methods against the baseline methods on noiseless images.

The performance is measured as the percentage of images where the lumen centerline was correctly detected, under the “80% correct” criterion.

**Figure 13:**
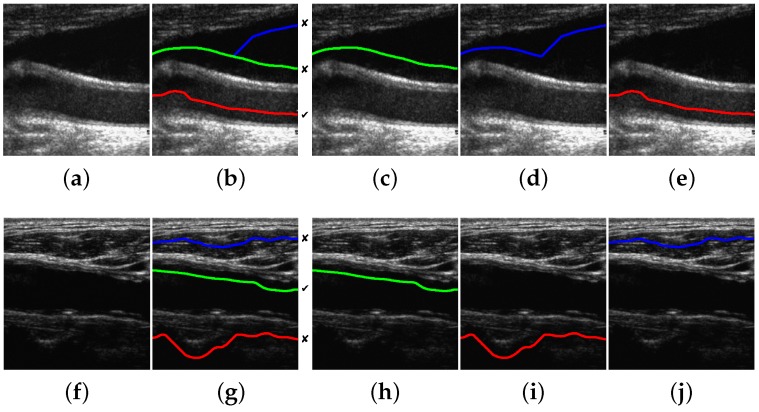
Examples of misdetections for the proposed method. From left to right: (**a**,**f**) input image; (**b**,**g**) detected candidates indicating which one is the correct lumen (✓); (**c**,**h**) symmetry-based selection; (**d**,**i**) intensity-based selection; and (**e**,**j**) contrast-based selection. The lumen centerlines selected by the Best-*Sym* [18] and the Bottom-most methods correspond to (c,h) and to (d,j), respectively.

The proposed method improves the results of the method described in [18] (Best-*Sym*) for noiseless images, when the symmetry or the positive contrast features are used. As in the case of classification results, the performance of the line features is slightly higher than that of the band features. However, when intensity features are used, the proposed method does not improve the results of the Best-*Sym* method. This implies that it would be better to simply select the candidate with the highest DP gain at the detection stage than to classify the candidates using the intensity information. At last, the results show that the negative contrast performs worse than the Bottom-most method. Thus, it is worse to select the candidates using the negative contrast than directly selecting the bottom-most candidate. Examples of the typical misdetection cases using symmetry, intensity and positive contrast features are shown in Figure 13.

**Figure 14:**
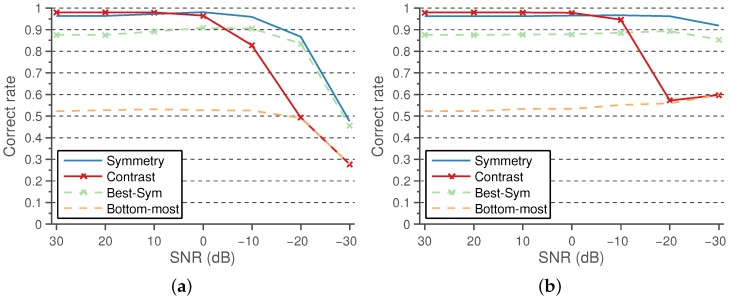
Comparison of lumen detection results under varying levels of artificial noise. The method performance rates of the proposed methods, using symmetry and positive contrast, are compared to the baseline methods. (**a**) Gaussian noise; (**b**) speckle noise.

The performance results using the symmetry and positive contrast line features, in comparison with the baseline methods, under varying levels of Gaussian and speckle noise are shown in Figure 14a,b, respectively.

As can be observed, both features are quite robust to reasonable levels of noise, the symmetry being the most robust feature, outperforming the robustness of Best-*Sym*.

### 4.4. Computational Efficiency

The proposed methods rely on a few convolutions and a dynamic programming algorithm with linear complexity in time. For the computation of the symmetry map, only three convolutions are necessary. The main overhead of the proposed methods in comparison to Best-*Sym* [18] is the computation of the contrast maps. The contrast maps only require two convolutions with Gaussian kernels, which are separable, and a point-to-point subtraction operation. Our MATLAB implementation of the proposed method takes 0.29±0.07 s/image, on a desktop computer equipped with an Intel Core i7 CPU at 3.07 GHz. Our implementation of the Best-*Sym* approach takes 0.23±0.06 s/image. Nevertheless, these implementations can be improved, for example, by using GPU and parallel computing optimizations, in order to achieve throughput rates of several frames per second.

## 5. Discussion

The candidate lumen detection algorithm, based on DP optimization of the symmetry map, is able to find a suitable candidate path going through the lumen region with high robustness. This behavior is also reproduced for the images in the dataset containing plaques, as illustrated in the examples of Figure 8i,m. Figure 8e also illustrates the robustness of the method to the presence of acoustic shadows. Furthermore, the proposed selection methods detect the true lumen for most images, especially when the positive contrast features and the position-based heuristics are used. A more detailed discussion on the factors that influence the performance is provided below.

The intensity does not provide a robust feature for the selection of lumen centerline candidates, mainly due to the presence of several dark regions at the image bottom, which appear in some carotid ultrasound images, as illustrated in Figure 13f. This can be caused by the attenuation of the ultrasound waves. This could be minimized by appropriately tuning the time gain compensation (TGC) parameters of the ultrasound systems to enhance the visualization of the high echogenicity characteristics of the connective tissue below the CCA. The other reason for the reduced robustness of intensity is the presence of lumen noise and artifacts, as in Figure 13a, which depend on the transducer positioning and the ultrasound gain parameters. The combination of these two effects makes the classifier unable to provide an appropriate threshold to discriminate the true lumen centerline from the other candidates that may appear below the artery. A possible solution to this is to use highly standardized acquisition parameters. However, this is not necessary, as the symmetry and contrast features do not have this parameter sensitivity and allow robust CCA detection. Furthermore, using candidate path selection with intensity features does not perform better than the Best-*Sym* [18], which is a simpler approach.

The small number of misdetections using the symmetry classifier occurs only for images with highly symmetric structures and patterns in the connective tissue below the CCA. There would be always the possibility to find structures with similar scales to the CCA, and in that case, the symmetry would not provide any useful discriminating information. Even though, the proposed method using symmetry classification outperforms the Best-*Sym* approach [18], regardless of the level of the artificially-added Gaussian or speckle noise. This indicates that the inclusion of the feature-based selection in combination with the position-based heuristic helps with reducing the misdetections, as is the aim of this work. In addition, the good results of the proposed method confirm that the symmetry map is a robust choice for candidate lumen centerline detection, even on the presence of high levels of noise, lumen artifacts and contrast variations.

The main source of misdetections using the contrast classifier is the poor estimation of the offset parameters for a few images. In some cases, as in the example of Figure 13f, the candidate lumen detector may find the lumen centerline slightly displaced from the center of the artery, and both the $G_{on}$ and the $G_{off}$ Gaussian filters may be displaced from the lumen and the adventitia regions, respectively. This problem may be addressed by increasing the $D_{max}$ parameter value for candidate detection, so that the symmetry measure tends to force a more centered candidate path on the wider arteries. However, even if the displacement issue was solved, the estimation of the adventitia position from the lumen centerline is only an approximation based on the average artery parameters that does not use any information derived from the image. A possible solution to address this issue would be to provide an image analysis algorithm that roughly estimates the adventitia from the lumen centerline and the image information, similar to that in [26], so that the computation of the lumen-to-wall contrast is more accurate. The higher number of misdetections of the negative contrast features seems to be due to the high echogenicity regions of the near wall being thinner than those of the far wall, so that the adverse effect of having set inappropriate DooG offset parameters for lumen-to-wall contrast computation is increased. Thus, in general, these results motivate the study of alternative methods to the global DooG parameter setting, with the aim of improving the lumen-to-wall contrast measurement.

On noiseless images, the highest performance is obtained using the proposed method with the positive contrast feature. The results improve both those of the Best-*Sym* approach and of the proposed method using the symmetry classifier. Nevertheless, as the noise levels are increased, the performance of the positive contrast drops to levels of the Bottom-most approach. It should be noted that the contrast feature is robust enough to reasonable levels of artificial noise, but it is worth discussing why this could happen with contrast and not with symmetry. The use of high levels of noise reduces the overall dynamic range of the image, which may affect the extrapolation capabilities of the thresholds selected by the logistic classifiers. As the symmetry is contrast normalized, the selected threshold extrapolates well to the presence of noise. This does not happen in the case of the contrast features, as the contrast is reduced for all of the candidates at high noise levels; the contrast difference between the true and false candidates is reduced; and thus, the selected threshold for noiseless images tend to not discard any of the lower contrast candidates. Therefore, the performance of the contrast features tends to those of the Bottom-most method, which does not discard any candidate before the position-based heuristic. This may be addressed normalizing the contrast feature in comparison with the overall image contrast.

## 6. Conclusions

This paper proposed new methods for the automatic detection of the CCA lumen centerline in B-mode ultrasound images. The goal was to improve the detection results of the symmetry-based method previously proposed in [18], by introducing advanced gating mechanisms to select the true CCA lumen from similar image structures. The major contributions are a robust candidate lumen detection and an advanced candidate selection, which takes advantage of relevant carotid artery features and position-based heuristics.

The candidate lumen detection has been demonstrated to be robust to noise. The symmetry map allows the use of a soft computing approach that avoids the common initial lumen segmentation step in other approaches, usually sensitive to lumen intensity variations and artifacts. Furthermore, the additional selection stages are able to consistently provide a correct lumen centerline among the detected candidate paths.

The lumen selection approach provides a general methodology that integrates feature-based criteria for defining a set of CCA candidates and a position-based heuristic that uses domain knowledge for the final true CCA detection. Furthermore, our approach allows one to incorporate additional single sources of information, as the intensity and contrast features. Finally, the results show that even a simple pattern recognition approach, as a single-feature logistic classifier, is suitable for threshold selection on noiseless images, which in combination with the image-wise normalization allows the trained model to extrapolate well to extremely noisy conditions, preserving noise robustness.

From the comparison of several features, it can be concluded that the positive contrast also provides excellent discriminating capabilities. Unlike the other features, it was identified that for the positive contrast, the very few misdetections were due to an inaccurate estimation of the DooG parameters on specific cases, which can be addressed in future improvements. Thus, future work may include the improvement of parameter estimation for lumen-to-wall contrast measurement, by incorporating an automatic adventitia candidate location method. However, in order to be able to obtain better estimates, the use of larger image datasets is encouraged. Even though, the proposed approach provides an excellent automatic lumen detection method that is robust to noise, lumen artifacts and ultrasound image contrast variations and that is able to deal with the presence of CCA-like structures.

## Figures and Tables

**Table 1:** Summary of the parameter estimation expressions and the resulting values.

	Parameter	Estimation	Value (mm)
Symmetry	$D_{max}$	${\hat{D}}_{max}$	15.8
	$D_{min}$	${\hat{D}}_{min}$	4.03
Peak detection	$\sigma_{peak}$	${\hat{D}}_{min}/4$	1.00
Contrast	$\sigma_{on}$	${\hat{D}}_{min}/2$	2.01
	$\sigma_{off}$	$({\hat{D}}_{max}-{\hat{D}}_{min})/4$	2.94
	*δ*	$({\hat{D}}_{max}+{\hat{D}}_{min})/4$	4.96
Band features	$R_{f}$	${\hat{D}}_{min}/2$	2.01

**Table 2:** Mean and standard deviation of features for the positive and negative lumen candidates, along with the *p*-value of Student’s *t*-test rejecting the equal distribution hypothesis.

Feature		Positive	Negative	*p*-Value
Line features	Symmetry	0.97 ± 0.02	0.95 ± 0.03	$<10^{-28}$
	Intensity	0.02 ± 0.03	0.05 ± 0.06	$<10^{-9}$
	Contrast +	0.19 ± 0.08	0.01 ± 0.05	$<10^{-124}$
	Contrast -	0.04 ± 0.05	0.11 ± 0.09	$<10^{-24}$
Band features	Symmetry	0.83 ± 0.03	0.77 ± 0.05	$<10^{-56}$
	Intensity	0.03 ± 0.03	0.07 ± 0.07	$<10^{-14}$
	Contrast +	0.17 ± 0.07	0.00 ± 0.05	$<10^{-121}$
	Contrast -	0.02 ± 0.05	0.09 ± 0.08	$<10^{-30}$

## Appendix

### Symmetry Map Computation

Similar to the Kovesi normalized symmetry measure [27], the local symmetry function for CCA detection has the form [18]:

(A1) $$Sym\left(x\right)=\frac{\left|\phi (x)\right|}{\pi }$$

where $x={\left(x,y\right)}^{⊤}$ denotes the image location, ϕ(x)∈[-π,π) is the local phase at x and ⊤ is the transpose operation. This symmetry measure linearly varies with phase deviation and has a maximum at ±π phases, which corresponds to the location of symmetry axes of dark regions.

The local phase information is derived from monogenic signal analysis [20]. The monogenic signal is an isotropic generalization of the analytic signal to two dimensions that uses the Riesz transform instead of the Hilbert transform. Since the Riesz transform is two-dimensional, the algebra of complex numbers is not sufficient to embed this generalized analytic signal. Instead, a subspace of H (quaternions) spanned by {1,i,j} is used [20]. The monogenic signal $f_{M}\left(x\right)$ of a given signal f(x) is given by:

(A2) $$f_{M}\left(x\right)=f\left(x\right)-\left(i,j\right)f_{R}\left(x\right)$$

where *i* and *j* denote the hyper-complex imaginary units and $f_{R}\left(x\right)$ corresponds to the Riesz transform of f(x), which in the frequency domain has the expression:

(A3) $$F_{R}\left(u\right)=\frac{iu}{\left||u|\right|}F\left(u\right)$$

where $u={\left(u_{1},u_{2}\right)}^{⊤}$ and F(u) is the Fourier transform of the input signal f(x).

The monogenic signal allows the estimation of the local amplitude, the local orientation and the local phase of the signal. The local phase ϕ(x) is obtained as:

(A4) $$\phi \left(x\right)=atan2(||f_{R}\left(x\right)||,f\left(x\right))$$

where atan2(·)∈[-π,π). The monogenic signal estimates the local phase in the direction of the locally strongest intrinsically one-dimensional signal. This phase is suitable for longitudinal B-mode CCA ultrasound image analysis, as their structures of interest are usually arranged in locally parallel layers.

Moreover, the analyzed signal f(x) is a band-pass filtered version of the input B-mode image. In [18], the band-pass filter was the radial log-normal filter, with a transfer function in the frequency domain defined by:

(A5) $$B_{\omega_{0},\kappa }\left(u\right)=\exp\left(-\frac{\left(\log(||u|\right|/\omega_{0}{))}^{2}}{2{\left(\log\left(\kappa \right)\right)}^{2}}\right)$$

where $\omega_{0}$ is the center frequency of the filter and *κ* is the parameter controlling the bandwidth. Log-normal filters allow arbitrarily large bandwidth with a zero DC component. The $\omega_{0}$ and *κ* parameters allow the selection of the size of the structures of interest. In [18], it was assumed that the possible CCA diameters were within the interval $\left[D_{min},D_{max}\right]$; thus, the filter parameters were selected according to the following equations:

(A6) $$\begin{array}{ccc} \omega_{0} & = & \frac{1}{\sqrt{D_{max}D_{min}}} \end{array}$$

(A7) $$\begin{array}{ccc} \kappa & = & e^{-\frac{1}{2\sqrt{2\log(2)}}\log\left(\frac{D_{max}}{D_{min}}\right)} \end{array}$$

so that the half-response cut-off frequencies of the filter correspond to $D_{max}^{-1}$ and $D_{min}^{-1}$.

The three components of the band-passed monogenic signal are computed in the frequency domain using the fast Fourier transform (FFT). In order to avoid periodization effects, a symmetrized border of width $D_{\max}$ is added at image boundaries before the FFT computation, and it is then removed in the filtered images.
